# A Complete Multimode Equivalent-Circuit Theory for Electrical Design

**DOI:** 10.6028/jres.102.029

**Published:** 1997

**Authors:** Dylan F. Williams, Leonard A. Hayden, Roger B. Marks

**Affiliations:** National Institute of Standards and Technology, Boulder, CO 80303

**Keywords:** conductor current, conductor representation, conductor voltage, electromagnetic modes, impedance matrix, modal representation, multiconductor transmission line

## Abstract

This work presents a complete equivalent-circuit theory for lossy multimode transmission lines. Its voltages and currents are based on general linear combinations of standard normalized modal voltages and currents. The theory includes new expressions for transmission line impedance matrices, symmetry and lossless conditions, source representations, and the thermal noise of passive multiports.

## 1. Introduction

This work extends the general waveguide circuit theory of Ref. [1] to multiple modes of propagation. The resulting equivalent-circuit theory mimics the low-frequency theory while rigorously accounting for loss. Unlike earlier treatments, the theory is constructed from the standard modal voltages and currents of Ref. [1], which are normalized so that the product of the modal voltage and current gives the power carried by a single mode in the absence of other modes in the guide [2] and so that they carry the conventional units volt and ampere. This approach easily and consistently generalizes the symmetry relations for reciprocal junctions reported in Refs. [1] and [3] and the noise results of Ref. [4], and maintains all of the conventional modal normalizations, units, and definitions. We present new conditions for lossless and passive devices, impedance matrix representations for multimode transmission lines, and Thevenin-equivalent voltage representations for the internal sources and thermal noise of a circuit, completing the multimode equivalent-circuit theory.

Maxwell’s equations are separable in the longitudinal and transverse directions of uniform waveguides and transmission lines. This leads to a natural description of the electromagnetic fields in the line in terms of the eigenfunctions of the two-dimensional eigenvalue problem. These eigenfunctions form a discrete set of forward and backward modes which propagate independently with an exponential dependence along their lengths; in open guides, this discrete set of modes is augmented by a continuous set of radiation modes. This modal description has a natural equivalent-circuit representation, even in the presence of loss [1]. In this representation each unidirectional mode is described by a modal voltage and current that propagate independently of those associated with the other modes of the line; this is the simplest equivalent-circuit representation of a lossy multimode transmission line from a physical point of view.

When a circuit can be partitioned into elements that communicate with each other through transmission lines supporting, in each case, only a single bidirectional mode, the modal description of Ref. [1] mimics closely the low-frequency theory, in which the complex power *p* is given by *v*_m_ *i*_m_*, where *v*_m_ is the modal voltage and *i*_m_ is the modal current. This allows the construction of a low-frequency equivalent-circuit analogy and the straightforward application of the methods of nodal analysis familiar to electrical engineers and commonly used for electrical design. To create the analogy, we specify reference planes far enough away from the ends of the lines interconnecting the circuit elements to ensure that only a single mode is present there. We then assign a node to each of these modes, setting the nodal voltages and currents equal to the modal voltages and currents. The normalization of Brews [2], which fixes the relationship between the modal voltages and currents, is used to ensure that the power in the actual circuit corresponds to that in the equivalent-circuit analogy.

The normalization of Brews leaves open the normalization of either the modal voltage or the modal current in each line, often chosen so as to simplify modeling of the circuit elements in the equivalent-circuit analogy. Typically the modal voltage is defined to correspond to the actual voltage between conductor pairs across which circuit elements are attached and the modal current is determined from the constraint on the power. It is also possible to define the modal current to correspond to the actual current in a particular conductor; in this case the modal voltage is determined from the constraint on the power.

Models of the embedded circuit elements can be further simplified in the equivalent-circuit analogy by representing them as an interior circuit connected to lines with lengths equal to those physically connected to the element. This approach results in simple lumped-element circuit models for the interior circuits that correspond closely to those predicted from physical models. While these models are not exact, they are extremely important for circuit design.

When multiple modes of propagation are excited in a transmission line, the total voltage across a given conductor pair will in general be a linear combination of all of the modal voltages and currents. Thus the circuit elements, which are usually connected between pairs of transmission-line conductors, will in general both excite and be excited by all of the modes propagating down the transmission line. As a result, the voltage across even the simplest of circuit elements, such as a resistor connected between a particular conductor pair, will not correspond to any one of the modal voltages but rather to a linear combination of all of them. This illustrates that the modal voltages and currents, which are associated with the modes rather than with the connection points of the circuit elements, do not correspond in even an approximate sense to those across or entering into the device terminals.

A number of authors, including those of Refs. [5], [6], [7], [8], and [9], have proposed models and equivalent-circuit theories for lossless multimode transmission lines. In Ref. [10] Jansen introduced the notion of a “partial power” characteristic impedance matrix for lossless coupled lines, which Tripathi and Lee [11] later extended to lossy coupled lines. Gardiol [12] considers loss in his development of an equivalent circuit theory and coupled transmission-line models but begins with assumptions of symmetric transmission-line representations.

Faché and De Zutter [13] proposed the first equivalent circuit theory applicable to general lossy coupled lines. It is based on power-normalized voltages and currents constructed from linear combinations of unnormalized modal voltages and currents. While these linear combinations may not correspond exactly to physical voltages between conductor pairs or physical currents in a particular conductor, Ref. [13] calls them the “conductor” or “circuit” voltages and currents. We will base our equivalent-circuit analogy on these power-normalized conductor voltages and currents.

**Fig. 1:**
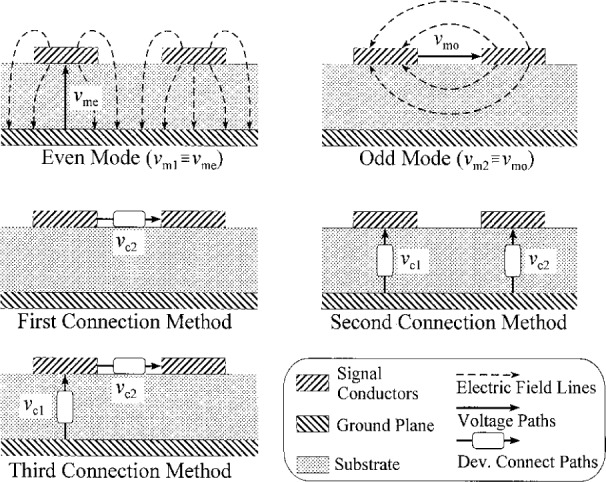
A symmetric microstrip line, its two dominant modes, and three methods of connecting devices between the conductors.

Figure 1 illustrates the equivalent-circuit theory of Ref. [13]. In this theory the appropriate choice of the conductor voltages and currents depends on the way in which circuit elements are connected to the transmission line. Figure 1 shows several ways in which discrete devices might be connected to a symmetric pair of microstrip lines. In the first, a single device is connected directly across the two signal conductors. The device will mainly excite the mode of the transmission line with odd electric field symmetry; its even mode is considered to be parasitic. Since the device communicates directly with one mode and parasitically with another, it would be appropriate to work directly with the modal equivalent-circuit representation of Ref. [1].

In the second connection method of Fig. 1, one device is connected between the left signal conductor and the ground plane, while the other is connected between the right signal conductor and the ground. Here each device excites both the even and odd modes of the transmission line. In this case it is easier to work with linear combinations of the modal voltages and currents, forming the first conductor voltage so that it corresponds to the integral of the electric field between the left signal line and the ground plane and the second so that it corresponds to that integral between the right signal line and the ground. Of course, there is some ambiguity here: different choices of paths between the conductors will give different voltages. This ambiguity, like its single mode counterpart, seems to be unavoidable.

For the third connection method of Fig. 1 yet even another choice of the conductor voltages is appropriate.

The conductor currents must be chosen as well. If we apply the same logic we used for our choice of conductor voltages to the second connection method of Fig. 1, we would select the first conductor current so that it is equal to the integral of the total magnetic intensity around a path enclosing the left signal conductor and the second conductor current equal to that integral around a path enclosing the right signal conductor. In fact, this choice suffers the same ambiguities as the choice of conductor voltage. For example, if the conductors are embedded in a lossy dielectric, some real current will flow there; it is no longer clear over which path we should integrate to define the conductor currents. Each new choice of integration path enclosing the conductors will change the currents in the conductor representation while leaving their voltages fixed. This simple example illustrates a difficulty with this strategy: the expression for the power will depend on the choice of the conductor voltages and currents and may not be compatible with that of the nodal low-frequency theory that the equivalent-circuit analogy is constructed to emulate. Expressions for the power are further complicated since the total power in the line is not generally the sum of the powers carried by each mode alone: examples of this behavior are discussed in Refs. [14] and [15].

In the single-mode case this difficulty is resolved by the power normalization of Brews [2], also used in Ref. [1]. There either the modal voltage or the modal current is fixed to correspond to an integral of the appropriate field quantity. The other is then determined so that the product of the voltage and the conjugate of the current gives the complex power.

Faché and De Zutter [13] developed a similar power-normalization procedure for the lossy multimode case; they picked either the conductor voltages or the conductor currents to correspond to the appropriate field integrals. As in the single-mode case, the undetermined quantity is found from a condition fixing the relation between the power and the conductor voltages and currents. This approach allows the construction of a useful equivalent-circuit analogy to which we can apply straightforward low-frequency nodal analysis methods.

Dhaene and De Zutter [16], Faché, Olyslager, and De Zutter [17], and Olyslager, De Zutter, and de Hoop [18] clarify and extend the theory of Ref. [13] and explore alternatives to the power normalization used there and in this work. However, none of these works includes all of the symmetry, noise, and other expressions needed to complete the equivalent-circuit theory. They also construct the conductor representation from unnormalized modal representations that do not result in the habitual units for the modal quantities and complicate their frequency dependence.

Here we examine the power-normalized conductor voltages and currents of Faché and De Zutter [13] constructed from general linear combinations of any number of the modal voltages and currents of Ref. [1], which carry conventional units and satisfy the power normalization of Brews [2]. This straightforward approach incorporates the advances of Refs. [1], [3], and [4] into the theory in a natural way and results in a complete equivalent-circuit theory for lossy multimode transmission lines that clearly illustrates and differentiates the modal and conductor representations. We develop concise definitions of impedance matrices and other circuit quantities and, for the first time, provide explicit means of incorporating multimode transmission lines in conductor representations via their impedance matrices: partial-power characteristic impedance matrices or symmetric per-unit-length representations are not required. We also present new symmetry and lossless conditions and expressions for the thermal noise of passive multiports.

## 2. Modal Description

We assume a time-harmonic dependence e^+^*^jωt^*, where *ω* is the real angular frequency, and that the transmission lines are uniform in *z*. These restrictions ensure that the electromagnetic boundary-value problem is separable in the longitudinal *z* coordinate and the transverse *x* and *y* coordinates. They also ensure that each line supports a countable set of discrete forward and backward modes [19] and, if the line is open, a continuous set of additional radiation modes [20]. All of these modes have, for some *γ*, an exponential *z* dependence e^±^*^γz^*. We will restrict our attention to finite or countable sets of modes excited in the line. In closed guides, we can account either for all of the modes or for just the subset of excited modes (usually the dominant modes) that enter into the problem. In open guides, the restriction of finite or countable sets of modes requires that we restrict ourselves to problems in which the continuous spectrum of radiation modes can be ignored.

We will also restrict our attention to lines constructed entirely of materials with isotropic permittivity and permeability, in which case the total transverse electric field ***E***_t_ and magnetic field strength ***H***_t_ in the line due to the excited modes with modal voltages *v*_m_*_k_* and *i*_m_*_k_* and transverse modal electric fields ***e***_t_*_k_* and magnetic field strengths ***h***_t_*_k_* can be written as

$$E_{t}\left(x,y,Z\right)=\sum_{k}\frac{\nu_{mk}\left(Z\right)}{\nu_{0k}}(e_{tk}\left(x,y\right)$$ (1)

and

$$H_{t}\left(x,y,Z\right)=\sum_{k}\frac{i_{mk}\left(Z\right)}{i_{0k}}h_{tk}\left(x,y\right),$$ (2)

where the sums span all of the excited modes in the line and we have added the dependence on the coordinates *x*, *y*, and *z* for clarity. Here the subscript m stands for “mode” and signifies the fact that the indicated quantity is associated with the modal, as opposed to the conductor, representation. The introduction of the normalizing factors *v*_0_*_k_* and *i*_0_*_k_* allows the *v*_m_*_k_* and *v*_0_*_k_* to have units of voltage, the *i*_m_*_k_* and *i*_0_*_k_* to have units of current, and the ***E***_t_, ***H***_t_, ***e***_t_*_k_*, and ***h***_t_*_k_* to have units appropriate to the fields. Appendix A shows that this is not so in the formulation of Ref. [13], which uses unnormalized modal voltages and currents, and presents conversions between all of the modal quantities in the conventional system of units used here and the unconventional system of units used in Ref. [13].

We restrict the normalizing voltage *v*_0_*_k_* and current *i*_0_*_k_* by

$$\nu_{0k}\;i_{0k}*=p_{0k}\equiv \int_{S}e_{tk}\times h_{tk}*\cdot Z\;dS,$$ (3)

where Re(*p*_0k_) ≥ 0. This normalizes the modal voltages and currents so that when only the *k*th mode is present, the complex power carried by the *k*th mode alone in the forward direction is given by *v*_m_*_k_ i*_m__k_*; this is the normalization used in Ref. [1] and corresponds to the power condition suggested by Brews [2].

The characteristic impedance of the *k*th mode is $Z_{0k}\equiv \nu_{0k}/i_{0k}={\left|\nu_{\theta_{k}}\right|}^{2}/p_{0k}*{\left|i_{0k}\right|}^{2}$; its magnitude is fixed by the choice of |*v*_0_*_k_*| or |*i*_0_*_k_*| while its phase is fixed by (3). With this definition, *Z*_0_*_k_* corresponds to the ratio of the modal voltage to the modal current in the line when only the kth forward mode is present, has units of ohms, and corresponds to accepted definitions [1].

The transmission line equations for the *k*th bidirectional mode are

$$\frac{d\nu_{mk}}{dZ}=-\left(\gamma_{k}\;Z_{0k}\right)i_{mk}\equiv -Z_{mk}\;i_{mk}\equiv -\left(R_{mk}+j\omega L_{mk}\right)i_{mk}$$ (4)

and

$$\frac{di_{mk}}{dZ}=-\left(\gamma_{k}\;/Z_{0k}\right)\nu_{mk}\equiv -Y_{mk}\;\nu_{mk}\equiv -\left(G_{mk}+j\omega C_{mk}\right)\nu_{mk},$$ (5)

where the *k*th mode has propagation constant ± *γk* and *L*_m_*_k_*, *R*_m_*_k_*, *C*_m_*_k_*, and *G*_m_*_k_* are real [1] and have the conventional units of inductance, resistance, capacitance, and conductance per unit length.

For a transmission line in which *n* modes propagate independently, we can express these transmission line equations in vector form as

$$\frac{dv_{m}}{dZ}=-Z_{m}i_{m}$$ (6)

and

$$\frac{di_{m}}{dZ}=-Y_{m}v_{m}.$$ (7)

Here ***v***_m_ and ***i***_m_ are column vectors of the modal voltages and currents of the various modes:

$$v_{m}\equiv {\left(\nu_{m1},\nu_{m2},\nu_{m3,\ldots }\right)}^{t}$$ (8)

and

$$i_{m}\equiv {\left(i_{m1},i_{m2},\;i_{m3,\ldots }\right)}^{t},$$ (9)

where the superscript t indicates the transpose. The diagonal matrices ***Z***_m_ and ***Y***_m_ of modal impedances and admittances per unit length of line are defined by

$$\begin{array}{r} Z_{m}\equiv \text{diag}\left(Z_{m1},\;Z_{m2},Z_{m3},\ldots \right)\\ =\text{diag}\left(\gamma_{1}Z_{01},\;\gamma_{2}\;Z_{02},\;\gamma_{3}\;Z_{03},\ldots \right) \end{array}$$ (10)

and

$$\begin{array}{r} Y_{m}\equiv \text{diag}\left(Y_{m1},\;Y_{m2},\;Y_{m3},\ldots \right)\\ =\text{diag}(\gamma_{1}/Z_{01},\;\gamma_{2}/Z_{02},\;\gamma_{3}/Z_{03},\ldots ). \end{array}$$ (11)

Equations (6) and (7) imply that

$$\frac{d^{2}v_{m}}{dZ^{2}}=Z_{m}\;Y_{m}\;v_{m}=\gamma^{2}\;v_{m}$$ (12)

and

$$\frac{d^{2}i_{m}}{dZ^{2}}=Y_{m}\;Z_{m}\;i_{m}=Z_{m}\;Y_{m}\;i_{m}=\gamma^{2}\;i_{m},$$ (13)

where the diagonal matrix ***γ*** is

$$\gamma \equiv \text{diag}(\gamma_{1},\;\gamma_{2},\;\gamma_{3},\ldots ).$$ (14)

**Fig. 2:**
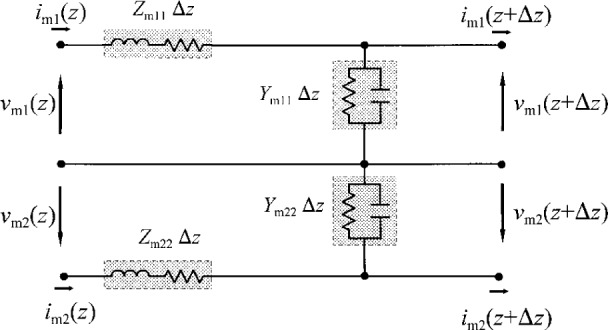
Modal equivalent-circuit model per unit length of transmission line for two modes of propagation.

Figure 2 shows the equivalent-circuit model for a multimode transmission line in the modal representation.

## 3. Conductor Representation

The modal representation, upon which the preceding discussion was based, is the simplest description of a multimode transmission line: its impedance and admittance matrices ***Z***_m_ and ***Y***_m_ per unit length are not only symmetric, but diagonal, and the voltages and currents corresponding to different modes are decoupled. However, we have already argued that this representation is not the most convenient for circuit design, where devices are connected between transmission line conductors. Following Ref. [13] we introduce the column vectors of conductor voltages ***v***_c_ and currents ***i***_c_, where the subscript c denotes the conductor or circuit parameters. However we define ***v***_c_ and ***i***_c_ to be arbitrary invertible linear transformations of ***v***_m_ and ***i***_m_, the conventionally normalized modal voltages and currents of Ref. [1]:

$$v_{c}\equiv M_{v}v_{m}$$ (15)

and

$$i_{c}\equiv M_{i}\;i_{m},$$ (16)

where both ***M***_v_ and ***M***_i_ are unitless.

Inserting these expressions into Eqs. (6) and (7) results in the transmission line equations for the conductor voltages and currents

$$\frac{dv_{c}}{dZ}=-Z_{c}i_{c}$$ (17)

and

$$\frac{di_{c}}{dZ}=-Y_{c}\;v_{c},$$ (18)

where the matrices of conductor impedances and admittances per unit length are defined by

$$Z_{c}\equiv R_{c}+j\omega L_{c}\equiv M_{v}\;Z_{m}\;M_{i}^{-1}$$ (19)

and

$$Y_{c}\equiv G_{c}+j\omega C_{c}\equiv M_{i}\;Y_{m}\;M_{v}^{-1},$$ (20)

where ***R***_c_, ***L***_c_, ***G***_c_, and ***C***_c_ are the transmission line’s matrices of resistances, inductances, conductances, and capacitances per unit length. Equations (15) and (16) imply that

$$\begin{array}{c} \frac{d^{2}v_{c}}{dZ^{2}}=Z_{c}\;Y_{c}\;v_{c}\\ =M_{v}\;Z_{m}\;Y_{m}\;M_{v}^{-1}v_{c}=M_{v}\;\gamma^{2}\;M_{v}^{-1}v_{c} \end{array}$$ (21)

and

$$\begin{array}{c} \frac{d^{2}i_{c}}{dZ^{2}}=Y_{c}\;Z_{c}\;i_{c}=\\ M_{i}\;Y_{m}\;Z_{m}\;M_{i}^{-1}i_{c}=M_{i}\;\gamma^{2}\;M_{i}^{-1}i_{c}. \end{array}$$ (22)

The matrices ***Z***_c_ ***Y***_c_ and ***Y***_c_ ***Z***_c_ are related to ***γ***^2^(= ***Z***_m_ ***Y***_m_ = ***Y***_m_ ***Z***_m_) by similarity transforms; thus all four matrices have the identical eigenvalues ***γ***^2^. ***M***_v_ diagonalizes ***Z***_c_ ***Y***_c_ and ***M***_i_ diagonalizes ***Y***_c_ ***Z***_c_. The equivalent-circuit model of Fig. 2 does not apply in the conductor representation because ***Z***_c_ and ***Y***_c_ are not in general diagonal.

## 4. Power

The total complex power *p* transmitted across a reference plane is given by the integral of the Poynting vector over the transmission-line cross section *S*:

$$\begin{array}{c} p=\int_{S}E_{t}\times H_{t}^{*}\cdot Z\;dS\\ =\sum_{j,k}\frac{\nu_{mj}(Z)}{\nu_{0j}}\frac{i_{mk}*\left(Z\right)}{i_{0k}*}\int_{S}e_{tj}\times \;h_{tk}*\cdot Z\;dS. \end{array}$$ (23)

This can be put into the form

$$p={i_{m}}^{⊤}\;X\;v_{m},$$ (24)

where the superscript ⊤ indicates the Hermitian adjoint (conjugate transpose) and the elements of the cross-power matrix ***X*** are

$$X_{kj}\equiv \frac{1}{\nu_{0j}\;i_{0k}*}\int_{S}e_{tj}\times \;h_{tk}*\cdot \;Z\;dS\;.$$ (25)

Reference [14] shows that the off-diagonal elements of ***X*** are often large in lossy quasiTEM multiconductor transmission lines near modal degeneracies. The diagonal elements of ***X*** are equal to 1 as a result of the normalization of (3), not used in Refs. [13], [16], [17], or [18].

Equation (24) becomes

$$p={i_{c}}^{⊤}{\left(M_{i}^{-1}\right)}^{⊤}X\;M_{v}^{-1}\;v_{c}$$ (26)

in the conductor representation.

## 5. Circuit Design

It is not our intention to determine the best choice of conductor voltages and currents for all situations: we have already argued that this choice is application dependent. However, we will formalize some of these choices here and in the next section and explore their implications.

### 5.1 Voltage

The *k*th row of ***M***_v_ determines the conductor voltage *v*_ck_. The *M*_v_*_kj_* can be chosen to set the conductor voltages *v*_c_*_k_* equal to the integral of the total electric field ***E***_t_ along any given path *l_k_*. The condition is

$$M_{vkj}=\frac{-1}{\nu_{0j}}\int_{l_{k}}e_{tj}\cdot \;d\;l\forall j⋗\nu_{0k}=-\int_{l_{k}}E_{t}\cdot dl,$$ (27)

where the symbol ⋗ means implies. Fixing all of the conductor voltages with Eq. (27) completely determines ***M***_v_. This voltage normalization is equivalent to that employed in Refs. [13], [16], [17], and [18].

### 5.2 Current

Likewise, we can force the conductor current *i*_c_*_k_* to correspond to the integral of the total magnetic field strength ***H***_t_ around a closed path *c_k_* by fixing the *k*th row of ***M***_i_. The condition is

$$M_{ikj}=\frac{1}{i_{0j}}\oint_{c_{k}}h_{tj}\cdot dl\forall j⋗i_{c_{k}}=\oint_{c_{k}}H_{t}\cdot dl.$$ (28)

Again, fixing all of the conductor currents with (28) completely determines ***M***_i_. This current normalization is equivalent to that employed in Refs. [13], [16], [17], and [18].

### 5.3 Complex Power

As we discussed in the introduction, one way to choose the conductor voltages and currents is to fix both ***M***_v_ and ***M***_i_ with Eqs. (27) and (28) for various choices of paths *l_k_* and *c_k_* and then to determine the complex power *p* from Eq. (26). However, Eq. (26) takes a form not found in the low-frequency nodal equivalent-circuit theory we want to emulate. This is because in conventional nodal analysis, which is used by all of the commercial circuit simulators of which these authors are aware, the power flowing into a circuit element is determined as $\sum_{k}\nu_{nk}\;i_{nk}*,$where *v*_n_*_k_* is the nodal voltage at the *k*th node, *i*_n_*_k_* is the nodal current flowing from that node into the circuit element, and the sum spans all of the nodes connected to the element. If we assign a node to each pair of conductor voltages and currents with the substitutions *v*_n_*_k_* = *v*_c_*_k_* and *i*_n_*_k_* = *i*_c_*_k_*, this simple expression does not agree with Eq. (26).

The expression for the power *p* in the conductor representation can be simplified by imposing the restriction $M_{i}^{⊤}\;M_{v}=X:$

$$M_{i}^{⊤}M_{v}=X⋗\;p=i_{c}^{⊤}\;v_{c}.$$ (29)

This form for *p*, which is also that of Refs. [13], [16], and [17], is useful because it mimics that of the low-frequency nodal equivalent-circuit theory. If we now assign a node to each pair of conductor voltages and currents and make the substitutions *v_nk_* = *v*_c_*_k_* and *i*_n_*_k_* = *i*_c_*_k_*, we find that the power *p* flowing into any circuit element corresponds exactly to that in the equivalent-circuit analogy; circuit simulators and computer aided design tools that determine power in the conventional way $\left(\text{i.e.},\;p={i_{n}}^{⊤}v_{n}\right)$ can be used without modification. We will show later that when this is done at all ports, it leads to some other conventional results, many of which are summarized in Table 1. Reference [15] shows that device modeling is simplified as well. We will call representations for which ${M_{i}}^{⊤}M_{v}=X$ ***M*** “power-normalized” conductor representations.

The restriction of Eq. (29) leaves open the determination of either ***M***_v_ or ***M***_i_ (but not both) by Eqs. (27) or (28). We could fix the conductor voltages, for example, to correspond to the integral of the total electric fields between the conductors to which we connect circuit elements by choosing the elements of ***M***_v_ with Eq. (27). Then ***M*** would be given by $M_{i}={\left(X\;M_{v}^{-1}\right)}^{⊤}={\left(M_{v}^{⊤}\right)}^{-1}\;X^{⊤}$. This is the multimode analogy of selecting the voltage-power normalization of characteristic impedance [1].

Alternatively, we could use Eq. (28) to fix the conductor currents. Then we would determine ***M***_v_ from $M_{v}={\left({M_{i}}^{⊤}\right)}^{-1}X$. This is the multimode analogy of selecting the current-power normalization of characteristic impedance. Either of these power normalizations results in the conductor voltages and currents of Ref. [13].

## 6. Determination of Modal Quantities from *Z*_c_ and *Y*_c_

The matrices of impedance and admittance parameters ***Z***_c_ and ***Y***_c_ in the power-normalized conductor representation can be used to determine ***M***_v_ and ***M***_i_, matrices which relate any modal quantity to its corresponding quantity in the conductor representation: we only need a single additional relation between each modal voltage and the conductor voltages or between each modal current and the conductor currents to fix the modal voltage or current paths. For example, since the columns of ***M***_v_ are proportional to the eigenvectors of ***Z***_c_***Y***_c_, we can fix them to within a constant. A single additional relation between one of the modal voltages and one of the conductor voltages then completely determines the corresponding columns of ***M***_v_. If the paths defining *v*_0_*_j_* and *v*_c_*_k_* are equal, for example, *M*_v_*_kj_* must be equal to one, completely defining the *k*th column of ***M***_v_.

The columns of ***M***_i_ are proportional to the eigenvectors of ***Y***_c_***Z***_c_, which also fixes them to within a constant: the columns of ***M***_i_ could also be fixed to within a constant from Eqs. (19) or (20). Equation (29) adds the additional constraint required to completely determine the columns of ***M***_i_, since it implies that the product of each column of ***M***_v_ and the complex conjugate of the corresponding column of ***M***_i_ must be equal to a diagonal element of ***X***, all of which are equal to 1.

Finally, the propagation constants *γ_j_* are the eigenvalues of ***Z***_c_***Y***_c_, completing the modal description.

Relations between the modal and conductor voltages can be used in place of relations between the modal and conductor currents in this procedure. This procedure forms the basis for the calibration and measurement algorithms described in [15], [21], and [22].

## 7. Impedance Matrix

**Fig. 3:**
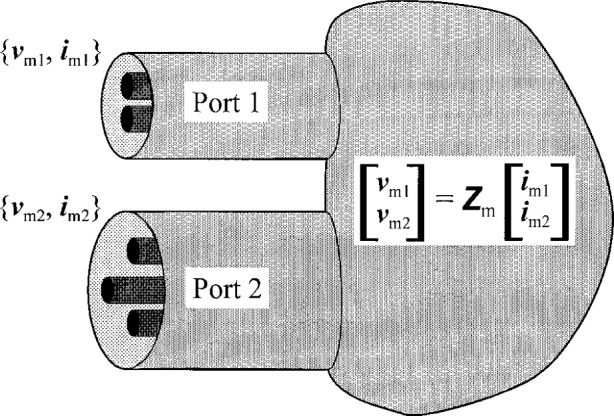
Linear network connecting two multimode transmission lines.

Figure 3 shows a linear network connecting two multimode transmission lines. We define the modal voltage vector ***v***_m_ and current vector ***i***_m_ by

$$v_{m}\equiv \left[\begin{array}{c} v_{m1}\\ v_{m2}\\ \vdots \end{array}\right];\;i_{m}\equiv \left[\begin{array}{c} i_{m1}\\ i_{m2}\\ \vdots \end{array}\right],$$ (30)

where ***v***_m_*_k_* and ***i***_m_*_k_* are the modal voltage and current vectors at port *k*. They are related by the network’s modal impedance matrix ***Z***_m_:

$$v_{m}=Z_{m}\;i_{m}.$$ (31)

We define the network’s conductor impedance matrix ***Z***_c_ as

$$Z_{c}\equiv M_{v}\;Z_{m}\;M_{i}^{-1},$$ (32)

where ***M***_v_ and ***M***_i_ are the block diagonal matrices

Mv≡[Mv1   Mv2   ⋱];Mi≡[Mi1   Mi2   ⋱], (33)

and the matrices ***M***_v_*_k_* and ***M***_i_*_k_* are the ***M***_v_ and ***M***_i_, respectively, for the transmission line at port *k*. These definitions imply that

$$v_{c}=Z_{c}\;i_{c},$$ (34)

where ***v***c and ***i***c are defined analogously to ***v***m and ***i***m.

## 8. Impedance Matrix of a Multimode Transmission Line

The modal impedance matrix ***Z***_mt_ of a section of multimode transmission line of length *l*_0_ is

$$Z_{\text{mt}}=\left[\begin{array}{cc} Z_{0}\;\coth\left(\gamma l_{0}\right) & Z_{0}\;\sinh{\left(\gamma l_{0}\right)}^{-1}\\ Z_{0}\;\sinh{\left(\gamma l_{0}\right)}^{-1} & Z_{0}\;\coth\left(\gamma l_{0}\right) \end{array}\right],$$ (35)

where *Z*_0_ ≡ diag(*Z*_0_*_j_*), coth(*γl*_0_) ≡ diag(coth(*γ_j_l*_0_)), and sinh(*γl*_0_)^−1^ ≡ diag(1/sinh(*γ_j_l*_0_)) are diagonal because each mode propagates independently down the line.

Equation (32) shows that the conductor impedance matrix ***Z***_ct_ of a section of multimode transmission line of length *l*_0_ is

$$Z_{\text{ct}}=\left[\begin{array}{cc} M_{v}Z_{0}\;\coth\left(\gamma l_{0}\right)M_{i}^{-1} & M_{v}Z_{0}\;\sinh\left(\gamma l_{0}\right)M_{i}^{-1}\\ M_{v}Z_{0}\;\sinh\left(\gamma l_{0}\right)M_{i}^{-1} & M_{v}Z_{0}\;\coth\left(\gamma l_{0}\right)M_{i}^{-1} \end{array}\right].$$ (36)

We have already seen that the matrices of impedance and admittance parameters ***Z***_c_ and ***Y***_c_, in addition to a single relation between each modal voltage and the conductor voltages or between each modal current and the conductor currents, can be used to determine *γ*, ***M***_v_, and ***M***_i_. It is then possible to find ***Z***_m_ and ***Y***_m_, and thus ***Z***_0_ and ***Z***_ct_, from ***Z***_c_ and ***Y***_c_.

Unlike ***Z***_0_coth(*γl*_0_) and ***Z***_0_sinh(*γl*_0_) ^−1^, the matrices $M_{v}\;Z_{0}\coth\left(\gamma_{0}\right)M_{i}^{-1}$ and $M_{v}\;Z_{0}\sinh{\left(\gamma l_{0}\right)}^{-1}M_{i}^{-1}$ are not diagonal; here again we see that the modal description will provide the simplest view of multimode transmission line behavior. Nevertheless, Eq. (36), which is useful in frequency-domain circuit simulations, provides a straightforward way to incorporate multimode transmission lines in the power-normalized conductor representation when ***Z***_c_ and ***Y***_c_ are asymmetric.

## 9. Reciprocal Junctions

References [1] and [3] develop a symmetry relation for the impedance matrix of a reciprocal junction (a passive junction that is composed only of materials with linear symmetric permittivity and permeability tensors) connecting transmission lines, each of which supports a single mode of propagation. This relation can be extended easily to the impedance matrix of a reciprocal junction connecting multimode transmission lines within the context of this theory. When none of the modes at any given port of a closed guide are degenerate $\left(\gamma_{j}^{2}\neq \gamma_{k}^{2}\;\text{for}\;j\;\neq k\right)$, then the basis fields at that port satisfy the orthogonality condition [19]

$$\int_{S}e_{tj}\times \;h_{tk}\cdot Z\;dS=0\;\left(j\neq k\right).$$ (37)

In open guides, a similar orthogonality condition is satisfied by the continuous spectrum of radiation modes [20], [23]. These orthogonality conditions allow the arguments of Refs. [1] and [3] to be applied directly, with the result that, for reciprocal junctions,

$$Z_{m}^{t}=W_{m}\;Z_{m}\;W_{m}^{-1},$$ (38)

where the diagonal matrix ***W***_m_ is defined by

Wm≡[Wm1   Wm1   ⋱], (39)

and where the ***W***_m_*_k_*, defined by

$$W_{mk}\equiv \text{diag}\left(\frac{1}{\nu_{0j}i_{0j}}\int_{S_{k}}e_{tj}\times \;h_{tj}\cdot Z\;dS\right),$$ (40)

are diagonal matrices of the reciprocity factors of Appendix D of Ref. [1] for the modes at port *k*. References [3] and [24] calculate elements of ***W***_m_ for some waveguides and Appendix B gives some cases for which ***W***_m_ is the identity matrix ***I***.

Substituting Eq. (32) into Eq. (38) gives the symmetry condition

$$Z_{c}^{t}=W_{c}\;Z_{c}{\left(W_{c}^{t}\right)}^{-1}$$ (41)

for a reciprocal junction in the conductor representation, where

$$W_{c}\equiv {\left(M_{i}^{t}\right)}^{-1}\;W_{m}\;M_{v}^{-1}.$$ (42)

The symmetry conditions for the impedance matrices of one-port terminations can be derived as special cases of Eq. (38) and (41).

## 10. Symmetric Impedance and Admittance Matrices

**Fig. 4:**
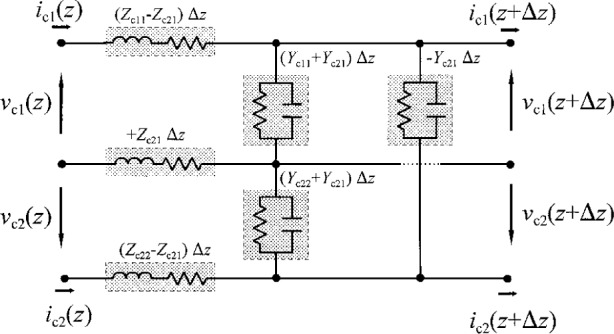
Conductor equivalent-circuit model per unit length for a two-mode transmission line with ***Z***_c_ and ***Y***_c_ symmetric.

Olyslager, De Zutter, and de Hoop in Ref. [18] present conductor representations in which ***Z***_c_ and ***Y***_c_ are always symmetric, in which case the equivalent-circuit description per unit length transmission line of Fig. 4 applies. There is, in fact, a hierarchy of symmetry conditions, which are sometimes treated as being equivalent in the literature.

Appendix C examines the weakest of these conditions, which simply ensures that, in the absence of degenerate modes $\left(\gamma_{j}^{2}\neq \gamma_{k}^{2}\;\text{for}\;j\;\neq k\right)$, ***Z***_c_ and ***Y***_c_ are symmetric. The requirement is that $M_{v}^{t}\;M_{i}$is diagonal:

$$M_{v}^{t}\;M_{i}\;\text{diagonal}\Leftrightarrow Z_{c}=Z_{c}^{t};\;Y_{c}=Y_{c}^{t},$$ (43)

where the symbol ⇔ means equivalent.

Appendix D examines two stronger conditions that ensure that the impedance matrices of passive junctions composed entirely of reciprocal materials are symmetric; it shows that the condition ensuring symmetry of all passive junctions embedded in a given line is

$$M_{v}^{t}\;M_{i}=\alpha \;W_{m}\Leftrightarrow W_{c}=\alpha I\Leftrightarrow Z_{c}=Z_{c}^{t},$$ (44)

where *α* is a scalar and ***Z***_c_ is the impedance matrix of *any* passive reciprocal junction embedded in the line. Appendix D also shows that there is a stronger condition that not only ensures that these impedance matrices are symmetric, but that the impedance matrices of junctions connecting the lines other lines satisfying the same condition are symmetric as well. It is

$$M_{v}^{t}\;M_{i}=\;W_{m}\Leftrightarrow W_{c}=I\Leftrightarrow Z_{c}=Z_{c}^{t},$$ (45)

where ***Z***_c_ is the impedance matrix of *any* passive reciprocal junction embedded in the line or connecting it to any other line with ***W***_c_ = ***I***. This condition is particularly interesting because it is the analog of the condition of Eq. (29): choosing either the conductor voltages or currents with Eq. (27) or Eq. (28) and applying the condition in Eq. (29) completely determines both ***M***_v_ and ***M***_i_. It is also a natural choice for ***W***_c_ in lossless lines.

All of these conditions require at least $M_{v}^{t}\;M_{i}$ diagonal, which is not always compatible with the condition $M_{i}^{⊤}\;M_{v}=X$ of the power-normalized conductor representation [18]. Thus enforcing any of these symmetry conditions will, at least in some cases, require abandoning the analogy with low-frequency nodal equivalent-circuit theory, in which $p=i_{n}^{⊤}\;v_{n}$.

At first glance a lack of these conventional symmetry conditions in the power-normalized conductor representation may seem problematic. However, in all lossless lines, for which the cross-power matrix ***X*** and modal reciprocity matrix ***W***_m_ are the identity, the conditions of Eqs. (29) and (45) are compatible (see Appendix B). We will also show that for the lossy quasi-TEM lines we examine in Secs. 14 and 15 that ***W***_c_ is almost exactly equal to the identity matrix in the power-normalized conductor representation and so nearly satisfy the strongest of these symmetry conditions.

If in the power-normalized conductor representation we cannot achieve even the weakest condition represented by Eq. (43), with the result that ***Z***_c_ and ***Y***_c_ are asymmetric, we can still include a section of line in the power-normalized conductor representation by way of its conductor impedance matrix, concisely expressed by Eq. (36).

## 11. Passive and Lossless Junctions

The real power *P* flowing into a passive junction must always be zero or positive for any external excitation. That is, for any passive junction,

P=Re(p)=Re(im⊤Xvm)=Re(im⊤XZmim)=12im⊤(XZm+(XZm)⊤)im≥0∀im, (46)

which is equivalent to the Hermitian matrix ***X Z***_m_ + (***X Z***_m_)^⊤^ being positive semidefinite [25]. For a lossless junction *P* = 0, which implies that ***X Z***_m_ + (***X Z***_m_)^⊤^ = 0 [4].

In the power-normalized conductor representation we obtain the conventional results: ***Z***_c_ + ***Z***_c_^⊤^ is positive semidefinite for passive circuits and ***Z***_c_ + ***Z***_c_^⊤^ = 0 for lossless circuits.

## 12. Thevenin-Equivalent Voltage Sources

The vector ${\hat{v}}_{m}$ of modal Thevenin-equivalent voltage sources of a linear network with impedance matrix ***Z***_m_ is defined by

$$v_{m}=Z_{m}i_{m}+{\hat{v}}_{m}.$$ (47)

While the vector ${\hat{v}}_{m}$ is general enough to describe electrically any linear sources within the network, the matrix ${\hat{v}}_{m}{{\hat{v}}_{m}}^{⊤}$ conveniently expresses the essential properties of the sources from an external point of view when their absolute phases are not of importance. Here the *j*th diagonal element of ${\hat{v}}_{m}{{\hat{v}}_{m}}^{⊤}$ is ${\left|{\hat{v}}_{mj}\right|}^{2}$ and its *jk*th off-diagonal element is ${\hat{v}}_{mj}\;{\hat{v}}_{mk}*$. These off-diagonal elements contain the relative phases of the sources in ${\hat{v}}_{m}$.

The Thevenin-equivalent sources in the conductor representation are ${\hat{v}}_{c}\equiv M_{v}\;{\hat{v}}_{m}$ and satisfy

$$v_{c}=Z_{c}\;i_{c}+{\hat{v}}_{c}.$$ (48)

The matrix ${\hat{v}}_{c}{{\hat{v}}_{c}}^{⊤}$ is related to ${\hat{v}}_{m}{{\hat{v}}_{m}}^{⊤}$ by

$${\hat{v}}_{c}{{\hat{v}}_{c}}^{⊤}=M_{v}\;{\hat{v}}_{c}{{\hat{v}}_{c}}^{⊤}\;{M_{v}}^{⊤}.$$ (49)

## 13. Thermal Noise

The thermal noise properties of a network are conveniently expressed in the modal representation by the matrix $<{\hat{v}}_{m}{{\hat{v}}_{m}}^{⊤}>$ [4], where the brackets indicate that we have taken the spectral density. The *j*th diagonal element of, $<{\hat{v}}_{m}{{\hat{v}}_{m}}^{⊤}>$ is $<{\left|{\hat{v}}_{mj}\right|}^{2}>$ the Fourier transform of the auto-correlation of ${\hat{v}}_{mj}$, while the *jk*th off-diagonal element is $<{\hat{v}}_{mj}{\hat{v}}_{mk}*>$, the Fourier transform of the cross-correlation of ${\hat{v}}_{mj}$ and ${\hat{v}}_{mk}$ [26], [27]. These frequency-domain quantities may be used to determine noise power in a circuit from straightforward ac analyses in which the noise sources are replace with nonrandom sinusoidal sources [26].

Reference [4] gives an expression for $<{\hat{v}}_{m}{{\hat{v}}_{m}}^{⊤}>$ for a passive network embedded deeply enough in a closed waveguide so that all but the dominant modes have decayed at the reference planes where we define the voltages and currents. The expression is

$$<{\hat{v}}_{m}{{\hat{v}}_{m}}^{⊤}>=2\frac{hf}{e^{hf/kT}-1}[Z_{m}Q+{\left(Z_{m}Q\right)}^{⊤}],$$ (50)

where ***V***_m_ contains all of the dominant modal voltages, *f* is the frequency, *k* is the Boltzmann constant, *h* is the Planck constant, *T* is the absolute temperature of the system, and ***Q*** = ***W***_m_^−1^ ***X***^t^(***W***_m_^⊤^)^−1^. Reference [4] presents practical lines in which ***Q*** differs significantly from the identity, which we will study further in Sec. 15.

Equation (50) in the conductor representation is

$$<{\hat{v}}_{c}{{\hat{v}}_{c}}^{⊤}>=2\frac{hf}{e^{hf/kT}-1}[Z_{c}M_{i}Q{M_{v}}^{⊤}+{\left(Z_{c}M_{i}Q{M_{v}}^{⊤}\right)}^{⊤}].$$ (51)

Equation (51) takes the conventional form when **W**_c_ is the identity matrix. In that case, ***M***_i_^t^ ***M***_v_ = ***M***_v_^t^ ***M***_i_ = **W**_m_ and in the power-normalized conductor representaiotn we have $Q=M_{i}^{-1}{\left(M_{v}^{⊤}\right)}^{-1}$, and Eq. (51) gives the conventional result

$$\begin{array}{c} W_{c}=I\;\text{and}\;M_{i}^{⊤}M_{v}=X\Rightarrow \\ <{\hat{v}}_{c}{{\hat{v}}_{c}}^{⊤}>=2\frac{hf}{e^{hf/kT}-1}[Z_{c}+{Z_{c}}^{⊤}]. \end{array}$$ (52)

## 14. Symmetric Coupled Microstrip Lines

Table 2 illustrates the application of this theory to the coupled symmetric microstrip lines of Fig. 1, for which cross-power matrix ***X*** is the identity due to symmetry (see Appendix B). The first row of the table lists the ***M***_v_ obtained by applying Eq. (27) to the paths appropriate to the three connection methods of Fig. 1. For the first connection method, ***M***_v_ is simply the identity matrix and the conductor voltages are equal to the modal voltages.

**Fig. 5:**
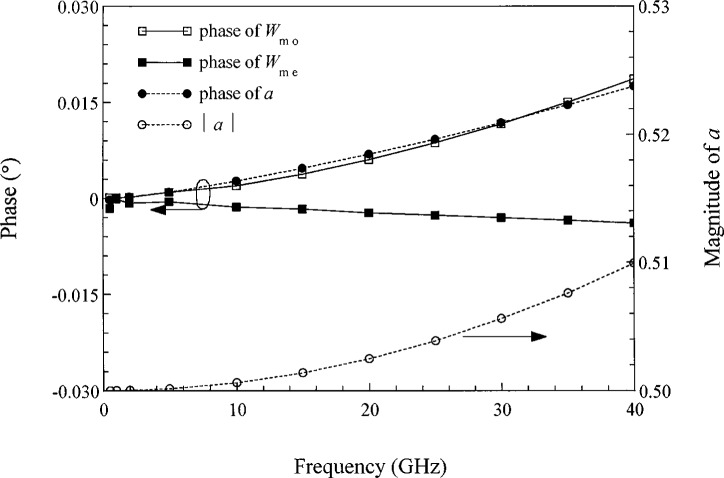
Modal parameters for the symmetric microstrip line of Fig. 1 with two 73 μm wide and 0.25 μm thick signal conductors separated by a gap of 50 μm. The 100 μm thick substrate has a relative dielectric constant of 12.9 and the substrate ground plane is 5 μm thick. The metal conductivities are 3.602 × 10^7^Ω^−1^ · m^−1^. Our calculations show that the magnitudes of *W*_me_ and *W*_mo_ depart from 1 by less than 10^−4^.

***M***_v_ for the second connection method of Fig. 1 reflects the fact that both modes impress voltages on the device connection paths. Here the even mode impresses the same voltage across the two connection paths. Since the path defining the even mode voltage corresponds to that over which the devices are connected, 1’s appear in the first column of ***M***_v_. The odd mode, on the other hand, impresses voltages of opposite phase on the two connection paths, and the odd mode voltage path does not correspond to the device connection path. We defined *a* to be the ratio of the voltage impressed by the odd mode between the ground plane and the right signal conductor and the modal voltage *v*_mo_ of the odd mode, which is defined as the total voltage between the two signal conductors. This accounts for the factors of ±*a* in the second column of ***M***_v_. Figure 5 plots the magnitude and phase of *a* calculated by the full-wave mode-matching method of Ref. [28] for a typical symmetric line and shows that in the low-frequency limit *a* is about one-half.

***M***_v_ for the third connection method is defined analogously. The values in the first row of ***M***_v_ are the same as those of the second method because the first connection path is the same in both cases. However, ***M***_v21_ = 0 because the even mode does not impress any voltage between the two signal conductors where the second device is connected, and ***M***_v22_ = 1 because the even mode and second connection paths coincide.

The table also lists the ***M***_i_, ***Z***_c_, ***Y***_c_, and ***W***_c_ obtained in the power-normalized conductor representation. Here ***M***_v_ and ***M***_i_ are dimensionless, have only a slight dependence on frequency, and are easily determined from straightforward arguments. This simplifies the determination of the conductor parameters from the standard modal parameters, which may often be found from conventional measurement methods or simple models. This convenient form of ***M***_v_ and ***M***_i_ is a result of beginning with the conventionally normalized modal voltages and currents of Ref. [1]. Note that the matrices corresponding to ***M***_v_ and ***M***_i_ in Ref. [13] carry the dimensions of voltage and current and, even in this symmetric example, will be highly frequency dependent (see Appendix A).

## 15. Asymmetric Coupled Microstrip Lines

**Fig. 6:**
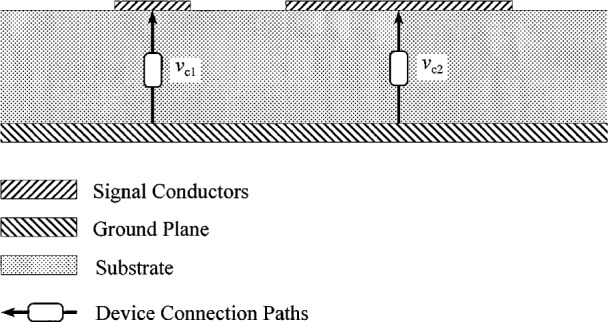
The asymmetric microstrip line and the method of connecting devices between the conductors studied here. The 30 mm wide signal conductor on the left is separated from the 200 μm wide signal conductor on the right by a 50 μm wide gap. The 100 μm thick substrate has a relative dielectric constant of 12.9. The 0.5 μm thick signal conductors and 5 μm thick ground plane have a conductivity of 3.602 × 10^7^Ω^−1^ · m^−1^.

Williams and Olyslager [14] show that the off-diagonal elements of ***X*** are large in lossy quasi-TEM multi-conductor transmission lines near modal degeneracies. Figure 6 shows the asymmetric lines used in Ref. [14] to illustrate this phenomena. These asymmetric coupled lines support two quasi-TEM dominant modes conventionally labeled the c and π modes. The c and π modes correspond to the even mode and the odd mode of the symmetric case, respectively.

**Fig. 7:**
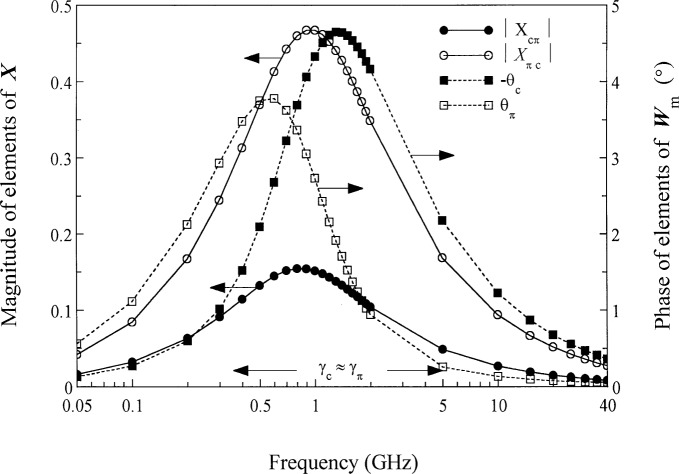
Modal parameters for the asymmetric coupled lines of Fig. 6. the frequencies at which Im(*γ*_c_−*γ_π_*) = 0 and at which |*γ*_c_−*γ_π_*|/*β*_0_ reaches a minimum define the frequency range labeled *γ*_c_ ≈ *γ_π_* in the figure.

**Fig. 8:**
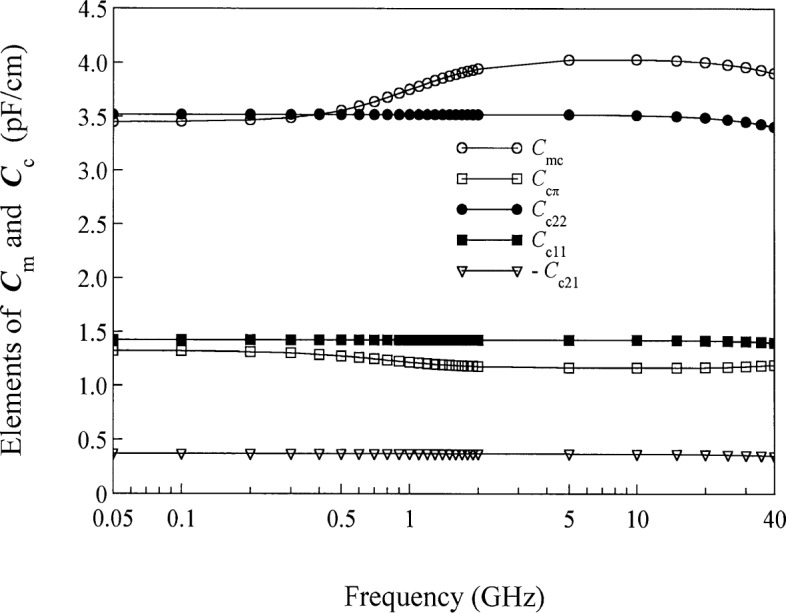
The elements of the capacitance matrices ***C***_m_ and ***C***_c_ for the coupled lines of Fig. 6. The calculations show that the elements of ***G***_m_ and ***G***_c_ are small and that |*C*_c12_−*C*_c21_| ≤ 2.0 × 10^−4^ pF/cm.

Appendix F gives a special form for ***X*** and ***W***_m_ appropriate for the c and *p* modes of the structure of Fig. 6. That form is

$$X=\left[\begin{array}{cc} 1 & -j\left|X_{c\pi }\right|e^{-j(\theta_{c}-\theta_{\pi })/2}\\ +j\left|X_{\pi c}\right|e^{j(\theta_{c}-\theta_{\pi })/2} & 1 \end{array}\right]$$ (53)

and

$$W_{m}=\sqrt{1-\left|X_{c\pi }X_{\pi c}\right|}\left[\begin{array}{cc} e^{j\theta c} & 0\\ 0 & e^{j\theta_{\pi }} \end{array}\right],$$ (54)

where we have chosen the appropriate signs in the general expressions given in the appendix for this example. Figure 7 plots the terms which appear in Eqs. (53) and (54) and shows that, despite the quasi-TEM nature of the lines and the lossless dielectric, the modal representation is quite complicated. Reference [14] shows that this can be attributed to a near degeneracy in the modal propagation constants. The complicated behavior of the modal representation is also reflected in variations of the modal capacitances *C*_mc_ and *C*_mπ_, which Fig. 8 shows change significantly with frequency.

The lines of Fig. 6 are simply described in the power-normalized conductor representation. Figure 8 shows that the elements of ***C***_c_ are approximately constant, as would be expected given the lossless substrate. Unlike *C*_mc_ and *C*_mπ_, the elements of ***C***_c_ are only weakly dependent on the metal loss.

**Fig. 9:**
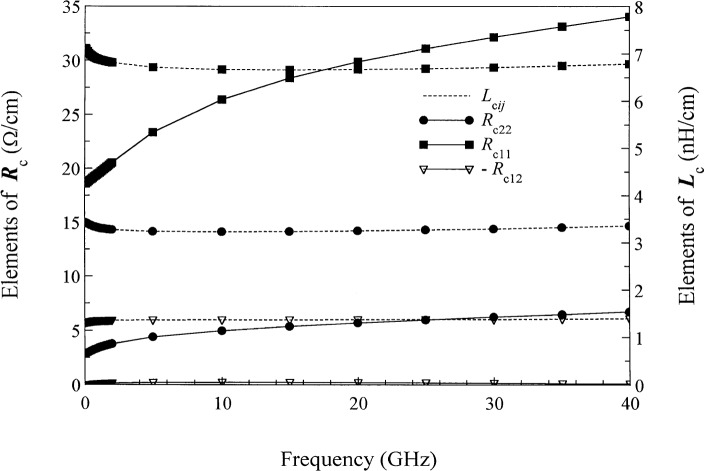
The elements of ***R***_c_ and ***L***_c_ for the coupled lines of Fig. 6. The calculations show that |*R*_c12_−*R*_c21_| ≤ 0.1 Ω/cm and |*L*_c12_−*L*_c21_| ≤ 10^−3^ nH/cm.

Figure 9 shows the elements ***R***_c_ and ***L***_c_ in the power-normalized conductor representation. They display the behavior typical of conductors at microwave frequencies: the elements of ***R***_c_ increase slowly with frequency as the fields are expelled from the metals and depend strongly on the metal loss while the elements of ***L***_c_ increase slightly at very low frequencies where the fields penetrate deeply into the metals.

Our numerical calculations based on the full-wave analysis method of Ref. [28] show that the elements of ***W***_c_ in this case differed from those of the identity matrix by less than 5 × 10^−4^ below 40 GHz. This implies that the impedance matrix of passive reciprocal devices embedded in these transmission lines are very nearly symmetric and the transmission-line equivalent-circuit model of Fig. 4 is appropriate in the power-normalized conductor representation.

Although Ref. [4] showed that *Q* for this structure differs significantly from the identity, complicating the calculation of thermal noise in the modal representation, our calculations show that the matrix $M_{i}\;Q\;M_{v}^{⊤}$ of Eq. (51) is also almost exactly equal to the identity matrix ***I***. Thus in the power-normalized conductor representation the conventional Eq. (52) for the thermal noise of a passive termination embedded in these lines applies, as we would anticipate from the fact that ***W***_c_ is nearly equal to the identity matrix ***I*** in this case. Reference [15] shows that the power-normalized conductor representation also simplifies device models. These results illustrate the advantages of using the conductor rather than the modal description when the off-diagonal elements of ***X*** are large.

## 16. Conclusion

We have investigated a power-normalized multimode equivalent-circuit theory based on the normalized modal voltages and currents of Ref. [1]. Its conductor representation allows the construction of a nodal equivalent-circuit analogy suitable for electrical design. The theory incorporates all of the elements required for design with multimode transmission lines, including symmetry conditions for reciprocal terminations and junctions, explicit expressions for the impedance matrix of multi-mode transmission lines, source representations, and expressions for the thermal noise of passive multiports. We illustrated the theory with examples of both symmetric and asymmetric coupled lines.

## Figures and Tables

**Table 1:** Relations for the power-normalized conductor representation

Complex power	*p* = ***i***_m_^⊤^ ***X v***_m_	*p* = ***i***_c_^⊤^ ***v***_c_
***M***_v_	–	***M***_v_ = (***M***_i_^⊤^)^−1^ ***X***
***M***_i_	–	***M***_i_ = (***XM***_v_^−1^)^⊤^ = (***M***_v_^⊤^)^−1^ ***X***^⊤^
Reciprocal junction	***Z***_m_^t^ = ***W***_m_***Z***_m_***W***_m_^−1^	***Z***_c_^t^ = ***W***_c_***Z***_c_(***W***_c_^t^)^−1^
Passive circuit	***XZ***_m_+(***X Z***_m_)^⊤^ pos. semidef.	***Z***_c_ + ***Z***_c_^⊤^ pos. semidef.
Lossless circuit	***X Z***_m_ + (***X Z***_m_)^⊤^ = 0	***Z***_c_ + ***Z***_c_^⊤^ = 0

**Table 2:** Circuit parameters for symmetric coupled lines of Fig. 1 in the power-normalized conductor representation specified by Eqs. (27) and (29). Here ***X*** = ***I*** and *a*, which is plotted in Fig. 5, is approximately equal to 1/2 at low frequencies.

Parameter	First method	Second method	Third method
***M***_v_	$\left[\begin{array}{cc} 1 & 0\\ 0 & 1 \end{array}\right]$	$\left[\begin{array}{cc} 1 & -a\\ 1 & a \end{array}\right]$	$\left[\begin{array}{cc} 1 & -a\\ 0 & 1 \end{array}\right]$
***M***_i_	$\left[\begin{array}{cc} 1 & 0\\ 0 & 1 \end{array}\right]$	$\frac{1}{2}\left[\begin{array}{cc} 1 & \frac{-1}{a*}\\ 1 & \frac{1}{a*} \end{array}\right]$	$\left[\begin{array}{cc} 1 & 0\\ a* & 1 \end{array}\right]$
***Z***_c_	$\left[\begin{array}{cc} Z_{\text{me}} & 0\\ 0 & Z_{\text{mo}} \end{array}\right]$	$\left[\begin{array}{cc} Z_{\text{me}}+{\left|a\right|}^{2}Z_{\text{mo}} & Z_{\text{me}}-{\left|a\right|}^{2}Z_{\text{mo}}\\ Z_{\text{me}}-{\left|a\right|}^{2}Z_{\text{mo}} & Z_{\text{me}}+{\left|a\right|}^{2}Z_{\text{mo}} \end{array}\right]$	$\left[\begin{array}{cc} Z_{\text{me}}+{\left|a\right|}^{2}Z_{\text{mo}} & -aZ_{\text{mo}}\\ -a*Z_{\text{mo}} & Z_{\text{mo}} \end{array}\right]$
***Y***_c_	$\left[\begin{array}{cc} Y_{\text{me}} & 0\\ 0 & Y_{\text{mo}} \end{array}\right]$	$\frac{1}{4}\left[\begin{array}{cc} Y_{\text{me}}+\frac{1}{{\left|a\right|}^{2}}Y_{\text{mo}} & Y_{\text{me}}-\frac{1}{{\left|a\right|}^{2}}Y_{\text{mo}}\\ Y_{\text{me}}-\frac{1}{{\left|a\right|}^{2}}Y_{\text{mo}} & Y_{\text{me}}+\frac{1}{{\left|a\right|}^{2}}Y_{\text{mo}} \end{array}\right]$	$\left[\begin{array}{cc} Y_{\text{me}} & aY_{\text{me}}\\ a*Y_{\text{me}} & Y_{\text{mo}}+{\left|a\right|}^{2}Y_{\text{me}} \end{array}\right]$
***W***_c_	$\left[\begin{array}{cc} W_{\text{me}} & 0\\ 0 & W_{\text{mo}} \end{array}\right]$	$\frac{1}{2}\left[\begin{array}{cc} W_{\text{me}}+\frac{a*}{a}W_{\text{mo}} & W_{\text{me}}-\frac{a*}{a}W_{\text{mo}}\\ W_{\text{me}}-\frac{a*}{a}W_{\text{mo}} & W_{\text{me}}+\frac{a*}{a}W_{\text{mo}} \end{array}\right]$	$\left[\begin{array}{cc} W_{\text{me}} & aW_{\text{me}}-a*W_{\text{mo}}\\ 0 & W_{\text{mo}} \end{array}\right]$
$M_{i}QM_{v}^{t}$	$\left[\begin{array}{cc} {\left|W_{\text{me}}\right|}^{-2} & 0\\ 0 & {\left|W_{\text{mo}}\right|}^{-2} \end{array}\right]$	$\frac{1}{2}\left[\begin{array}{cc} {\left|W_{\text{me}}\right|}^{-2}+{\left|W_{\text{mo}}\right|}^{-2} & {\left|W_{\text{me}}\right|}^{-2}-{\left|W_{\text{mo}}\right|}^{-2}\\ {\left|W_{\text{me}}\right|}^{-2}-{\left|W_{\text{mo}}\right|}^{-2} & {\left|W_{\text{me}}\right|}^{-2}+{\left|W_{\text{mo}}\right|}^{-2} \end{array}\right]$	$\left[\begin{array}{cc} {\left|W_{\text{me}}\right|}^{-2} & 0\\ a*\left({\left|W_{\text{me}}\right|}^{-2}-{\left|W_{\text{mo}}\right|}^{-2}\right) & {\left|W_{\text{mo}}\right|}^{-2} \end{array}\right]$

**Table 3:** Renormalization table for unnormalized modal parameters

Modal parameters	This work (normalized system)	Corresponding quantity in Ref. [13]
Voltages and currents	***v***_m_, ***i***_m_	(***v***_0_)^−1^ ***v***_m_, (***i***_0_)^−1^ ***i***_m_
Normalization condition	$p_{0k}=\nu_{0k}i_{0k}^{*}$	$v_{0}^{\prime }=i_{0}^{\prime }={\left(1,1,\ldots \right)}^{t}$
Characteristic impedance	$Z_{0k}\equiv \frac{\nu_{0k}}{i_{0k}}=\frac{\left|\nu_{0k}\right|}{p_{0k}*}=\frac{p_{0k}}{{\left|i_{0k}\right|}^{2}}$	1
Transmission line parameters per unit length	***R***_m_ + *jω* ***L***_m_ ***G***_m_ + *jω* ***C***_m_	*γ* *γ*
Cross-power matrix	***X***	$i_{0}^{⊤}X\;v_{0}\equiv P^{t}$
Transformation matrices	***M***_v_, ***M***_i_	***M***_v_***v***_0_, ***M***_i_***i***_0_
Reciprocity matrix	***W***_m_	***v***_0_***i***_0_ ***W***_m_
Impedance matrix	***Z***_m_	***V***_0_^−1^***Z***_m_***i***_0_
Noise matrix *Q*	***Q***	***i***_0_^−1^***Q***(***v***_0_^⊤^)^−1^
Conductor parameters (equivalent in both systems)	***v***_c_, ***i***_c_, ***W***_c_, ***Z***_c_, …	***v***_c_, ***i***_c_, ***W***_c_, ***Z***_c_, …

**Table 4:** Renormalization table for power-normalized conductor parameters

Before normalization	After normalization
***v***_c_, ***i***_c_	*δ* ***v***_c_, (δ*)^−1^ ***i***_c_
***M***_v_, ***M***_i_	*δ* ***M***_v_, (δ*)^−1^***M***_i_
***Z***_c_, ***Y***_c_	*δ****Z***_c_*δ* *, (δ*)^−1^ ***Y***_c_ *δ*^−1^
***W***_c_, ***W***_c_	*δ* ***W***_c_*δ*^−1^, δ* ***W***_c_*δ*^−1^
***Z***_c_	*δ* ***Z***_c_ δ*
***M***_i_ ***Q M***_v_^⊤^	(δ*)^−1^ ***M***_i_ ***Q M***_v_^⊤^ δ*

## 17. Appendix A. Unnormalized Modal Voltages and Currents

Reference [13] forms conductor voltages and currents from linear combinations of unnormalized modal voltages ${v^{\prime }}_{m}$ and currents ${i^{\prime }}_{m}$. In those works the ${v^{\prime }}_{m}$ and ${i^{\prime }}_{m}$ are defined by

$$E_{t}=\sum_{k}v_{mk}^{\prime }e_{tk};H_{t}=\sum_{k}i_{mk}h_{tk}.$$ (55)

The ${v^{\prime }}_{m}$ and ${i^{\prime }}_{m}$ can be obtained from ***v***_m_ and ***i***_m_ by setting the *n*_0_*_k_* and *i*_0_*_k_* of this theory equal to 1, rather than applying the power condition $P=\nu_{0k}i_{0k}^{*}$ of Brews [2] and normalizations of Ref. [1]. Table 3 shows how this changes the various modal parameters and relations presented in this work. For example, the first line of the table shows that ${v^{\prime }}_{m}={\left(\nu_{0}\right)}^{-1}v_{m}$. The elements ***P*** given in the table are $P_{jk}\equiv \int_{S}e_{tj}\times h_{tk}^{*}\cdot Z\;d\;S$.

Reference [13] defines either the conductor voltages ***v***_c_' by

$$v_{ck}^{\prime }=-\int_{l_{k}}E_{t}\cdot dl$$ (56)

or the conductor currents $i_{c}^{\prime }$ by

$$i_{ck}^{\prime }=\oint_{c_{k}}H_{t}\cdot dl,$$ (57)

definitions that correspond to those of Eqs. (27) and (28) used here. Either of these definitions, in conjunction with the constraint $p={{i^{\prime }}_{c}}^{⊤}{v^{\prime }}_{c}$, results in ${v^{\prime }}_{c}=v_{c}$ and ${i^{\prime }}_{c}=i_{c}$. Thus we see that, although the modal parameters of the two systems are quite different, their conductor parameters are equivalent.

## 18. Appendix B. Symmetric and Power-Orthogonal Modes (*X* = *I*)

We can put the elements of the cross-power matrix ***X*** in the form

$$\begin{array}{l} X_{kj}=\frac{\nu_{0k}}{\nu_{0j}}\frac{\int_{S}\varepsilon *e_{tj}\cdot e_{tk}^{*}\;dS\int_{S}\mu h_{Zj}h_{Zk}^{*}\;dS}{\int_{S}\varepsilon *{\left|e_{tj}\right|}^{2}dS-\int_{S}\mu {\left|h_{Zk}\right|}^{2}dS}=\\ {\left(\frac{i_{0j}}{i_{0k}}\right)}^{*}\frac{\int_{S}\mu h_{tj}\cdot h_{tk}^{*}\;dS-\int_{S}\varepsilon *e_{zj}\cdot e_{tk}^{*}\;dS}{\int_{S}\mu {\left|h_{tk}\right|}^{2}dS-\int_{S}\varepsilon *{\left|e_{Zj}\right|}^{2}dS} \end{array}$$ (58)

by following the arguments of Appendix B of Ref. [1]. Equation (58) shows that ***X****_kk_* = ***X****_jj_* = 1 and that ***X****_kj_* = ***X****_jk_* = 0 if the *k*th and *j*th modes have opposite electric or magnetic field symmetries. For the symmetric coupled microstrip lines of Fig. 1, for example, ***X*** = ***I***.

We call the *k*th mode of a closed guide power orthogonal if ***X****_kj_* = ***X****_jk_* = 0 for *all* of the other modes *j* in the transmission line. If the *k*th mode is power orthogonal, then Ref. [4] shows that *W*_m_*_k_ W*_m_*_k_** = 1, and *W*_m_*_k_* can be set equal to 1 by suitable normalization of the phase of the ν_0_*_k_* or the *i*_0_*_k_* [1]. Lossless modes are power orthogonal.

When ***X*** = ***I***, the conditions represented by Eqs. (29) and (43) are compatible, so symmetric ***Z***_c_ and ***Y***_c_ can be achieved in the power-normalized conductor representation. However it is possible to show that simultaneous satisfaction of conditions represented by Eqs. (29) and (43) then requires that all of the elements in any given column of ***M***_v_ and in the same column of ***M***_i_ have the same phase. It is also possible to show that setting ***W***_c_ = ***I*** requires that the magnitudes of the diagonal elements of ***W***_m_ equal one and that the phase of the elements in the columns of ***M***_v_ and ***M***_i_ be set to one half the phase of the corresponding diagonal element of ***W***_m_.

## 19. Appendix C. Diagonality of $M_{v}^{t}$ *M*_i_ and Symmetry of *Z*_c_ and *Y*_c_

If *Z*_c_, the transmission line impedance matrix per unit length, is symmetric then

$$Z_{c}=M_{v}\;Z_{m}\;M_{i}^{-1}={\left(M_{i}^{-1}\right)}^{t}\;Z_{m}\;M_{v}^{t},$$ (59)

and

$$M_{i}^{t}\;M_{v}\;Z_{m}=Z_{m}\;M_{v}^{t}\;M_{i}={\left(M_{i}^{t}\;M_{v}\;Z_{m}\right)}^{t}.$$ (60)

Thus ***Z***_c_ is symmetric if and only if $M_{i}^{t}$ ***M***_v_ ***Z***_m_ is symmetric. Likewise, ***Y***_c_ is symmetric if and only if $M_{v}^{t}$ ***M***_i_ ***Y***_m_ is symmetric. Clearly, if $M_{i}^{t}$ ***M***_v_ is diagonal, then so are $M_{i}^{t}$ ***M***_v_ ***Z***_m_ and $M_{v}^{t}$ ***M***_i_ ***Y***_m_, and so ***Z***_c_ and ***Y***_c_ are symmetric.

The reverse is true for nondegenerate modes (modes for which *γ_j_*^2^ ≠ *γ_k_*^2^). First, ***Z***_c_ and ***Y***_c_ symmetric implies that both $M_{i}^{t}$ ***M***_v_ ***Z***_m_ and $M_{v}^{t}$ ***M***_i_ ***Y***_m_ are symmetric. The *jk*th element of $M_{i}^{t}$ ***M***_v_ ***Z***_m_ is

$${\left(M_{i}^{t}M_{v}Z_{m}\right)}_{jk}={\left(M_{i}^{t}M_{v}\right)}_{jk}Z_{mk}.$$ (61)

Thus $M_{i}^{t}$ ***M***_v_ ***Z***_m_ symmetric implies that ( $M_{i}^{t}$ ***M***_v_)_*jk*_ Z_m*k*_ = (***M***_i_ ***M***_v_)_*kj*_ Z_m*j*_. Likewise, $M_{v}^{t}$ ***M***_i_ ***Y***_m_ symmetric implies ( $M_{i}^{t}$ ***M***_v_)_*kj*_ *Y*_m*k*_ = ( $M_{i}^{t}$ ***M***_v_)_*jk*_ *Y*_m*j*_. Taking the product of these two equations and using $Z_{mk}Y_{mk}=\gamma_{k}^{2}$ gives ( $M_{i}^{t}$ ***M***_v_)_*jk*_ ( $M_{i}^{t}$ ***M***_v_)_*kj*_ (*γ*_j_^2^ − *γ_k_*^2^) = 0, which leads to either ( $M_{i}^{t}$ ***M***_v_)_*jk*_ = 0 or ( $M_{i}^{t}$ ***M***_v_)_*kj*_ = 0. Assume that ( $M_{i}^{t}$ ***M***_v_)_*j*k_ = 0. Then, from Eq. (60),

$$\begin{array}{c} {\left(M_{i}^{t}\;M_{v}\;Z_{m}\right)}_{kj}={\left(M_{i}^{t}\;M_{v}\right)}_{kj}\;Z_{m_{j}}\\ ={\left(M_{i}^{t}\;M_{v}\;Z_{m}\right)}_{jk}={\left(M_{i}^{t}\;M_{v}\right)}_{jk}\;Z_{m_{k}}=0, \end{array}$$ (62)

and so ( $M_{i}^{t}$ ***M***_v_)*_kj_* = 0 as well. A similar argument applies if we assume that it was ( $M_{i}^{t}$ ***M***_v_)*_kj_* that was 0. Thus $M_{i}^{t}$ ***M***_v_ is diagonal.

## 20. Appendix D. Symmetry of the Impedance Matrices of Reciprocal Junctions and Scalar *W*_c_

If ***W***_c_ is scalar, then Eq. (41) shows that the conductor impedance matrices ***Z***_c_ of all passive junctions constructed entirely of reciprocal materials embedded in it are symmetric.

If the conductor impedance matrix ***Z***_c_ of every passive junction constructed of reciprocal materials embedded in it is symmetric, then we can also show that ***W***_c_ is a scalar. From Eq. (41) we have

$$Z_{c}=Z_{c}^{t}=W_{c}\;Z_{c}{\left(W_{c}^{t}\right)}^{-1},$$ (63)

which implies that

$$Z_{c}\;W_{c}^{t}=W_{c}\;Z_{c}.$$ (64)

The *ij*th element of ***Z***_c_ $W_{c}^{t}$ is $\sum_{k}Z_{cik}\;W_{cjk}$ while the *ij*th element of ***W***_c_ ***Z***_c_ is $\sum_{k}W_{cik}\;Z_{ckj}$. Equating these two elements gives

$$\begin{array}{c} \left(W_{cjj}-W_{cii}\right)Z_{cii}+\sum_{k\neq j}W_{cjk}\;Z_{cik}\\ -\sum_{k\neq i}W_{cik}\;Z_{ckj}=0, \end{array}$$ (65)

which must hold true for the conductor impedance matrix of any junction constructed of reciprocal materials. For any given *i* ≠ *j*, Eq. (65) can only be true for all of these ***Z***_c_ if ***W***_c_*_jj_* − ***W***_c_*_ii_* and all of the terms ***W***_c_*_jk_* (*k* ≠ *j*) are independently equal to 0, which implies that ***W***_c_ is a scalar matrix.

Finally, if ***W***_c_ = *α****I***, where *α* is a scalar, then Eq. (42) shows that $M_{i}^{t}$ ***M***_v_ = *α****W***_m_.

If ***W***_c_ = *α****I***, then Eq. (41) shows that the conductor impedance matrices ***Z***_c_ of all junctions constructed entirely of reciprocal materials connecting the lines to other lines for which ***W***_c_ = *α****I*** will be symmetric. Likewise, if the conductor impedance matrices of all junctions constructed entirely of reciprocal materials connecting the lines to other lines for which ***W***_c_ = *α****I*** are symmetric, we must have ***W***c = *α****I***. This also holds true, of course, when ***W***_c_ = ***I*** in all the lines. This is the most convenient normalization since ***W***_c_ = ***I*** is the natural choice in lossless lines.

## 21. Appendix E. Renormalization Table

We have presented relations between modal and conductor quantities. Here we show how a renormalization of the conductor voltages and currents that preserves $M_{i}^{⊤}\;M_{v}=X$ affects the other conductor parameters. The second column shows the effect on the element in the first column after multiplying the voltage eigenvector by the matrix *δ* ≡ diag(*δ_q_*).

## 22. Appendix F. Form of *X* and *W*_m_ for Two Modes When *W*_c_ = *I*

Whenever it is possible to satisfy the power normalization condition of Eq. (29) and set ***W***_c_ = ***I*** simultaneously we have both $M_{i}^{T}\;M_{v}=X$ and $M_{i}^{t}\;M_{v}=W_{m}$. Thus we can write $X\;{W_{m}}^{-1}=M_{i}^{⊤}\;M_{v}\;{M_{v}}^{-1}{\left(M_{i}^{t}\right)}^{-1}=M_{i}^{⊤}{\left(M_{i}^{t}\right)}^{-1}$ and so ${\left(X\;W_{m}^{-1}\right)}^{*}X\;W_{m}^{-1}={\left(M_{i}^{⊤}{\left(M_{i}^{t}\right)}^{-1}\right)}^{*}M_{i}^{⊤}{\left(M_{i}^{t}\right)}^{-1}=I$.

Reference [4] shows that the condition ${\left(X\;W_{m}^{-1}\right)}^{*}X\;W_{m}^{-1}=I$ also holds if all the modes in the guide are accounted for.

If, for every mode *j* to which we have assigned a voltage and a current and for every mode *k* for which we have not, the cross-power integrals $\int e_{tj}\times \;h_{tk}*\cdot \;dS=\int e_{tk}\times \;h_{tj}*\cdot \;Z\;dS=0$, then as a corollary we have ${\left(X\;W_{m}^{-1}\right)}^{*}X\;W_{m}^{-1}=I$ as well.

When ${\left(X\;W_{m}^{-1}\right)}^{*}X\;W_{m}^{-1}=I$ and there are only two modes, which we will label the *c* and *π* modes, then ${\left(X\;W_{m}^{-1}\right)}^{*}={\left(X\;W_{m}^{-1}\right)}^{-1}$ and we can write ***X*** and ***W***_m_ as

$$X=\left[\begin{array}{cc} 1 & X_{c\pi }\\ X_{\pi c} & 1 \end{array}\right];W_{m}=\left[\begin{array}{cc} W_{\text{mc}} & 0\\ 0 & W_{m\pi } \end{array}\right],$$ (66)

which implies that

$$\begin{array}{l} \left[\begin{array}{cc} {\left(W_{\text{mc}}*\right)}^{-1} & X_{c\pi }*{\left(W_{m\pi }*\right)}^{-1}\\ X_{\pi c}*{\left(W_{\text{mc}}*\right)}^{-1} & {\left(W_{m\pi }*\right)}^{-1} \end{array}\right]=\\ \frac{W_{\text{mc}}W_{m\pi }}{1X_{c\pi }-X_{\pi c}}\left[\begin{array}{cc} {W_{m\pi }}^{-1} & -X_{c\pi }{W_{m\pi }}^{-1}\\ -X_{\pi c}{W_{\text{mc}}}^{-1} & {W_{\text{mc}}}^{-1} \end{array}\right]. \end{array}$$ (67)

Equating the diagonal terms in Eq. (67) implies that ${\left|W_{\text{mc}}\right|}^{2}={\left|W_{m\pi }\right|}^{2}=1-X_{c\pi }X_{\pi c}$, which implies that the product ***X***_cπ_ ***X***_πc_ is real and that we can write ***W***_mc_ and ***W***_mπ_ as $W_{\text{mc}}=\sqrt{1-X_{c\pi }X_{\pi c}}e^{j\theta_{c}}$ and $W_{m\pi }=\sqrt{1-X_{c\pi }X_{\pi c}}e^{j\theta_{\pi }}$. Equating the upper-right hand off-diagonal terms in Eq. (67) allows us to determine the phase of X_cπ_ in terms of θ_c_ and θ_π_ to within a factor of ±π, while equating the lower-left hand off-diagonal terms in Eq. (67) allows us to determine the phase of *X*_πc_. These constraints on the phase of *X*_cπ_ and *X*_πc_, in addition to the constraint that their product be real, result in the forms

$$X=\left[\begin{array}{cc} 1 & \pm j\left|X_{c\pi }\right|e^{j(\theta_{c}-\theta_{\pi })/2}\\ \pm j\left|X_{\pi c}\right|e^{j(\theta_{c}-\theta_{\pi })/2} & 1 \end{array}\right]$$ (68)

and

$$W_{m}=\sqrt{1-X_{c\pi }X_{\pi c}}\left[\begin{array}{cc} e^{j\theta_{c}} & 0\\ 0 & e^{j\theta \pi } \end{array}\right]$$ (69)

for ***X*** and ***W***_m_, respectively.
